# A Pilot Study of the Anthropometric Growth Pattern of the Human Auricle of the North Indian Region

**DOI:** 10.7759/cureus.76085

**Published:** 2024-12-20

**Authors:** Parvez Husain, Shaheen K Ahmed, Shikhar Saxena, Benazeer Husain, Aditya Jain, Aravind Madhwacharya

**Affiliations:** 1 Department of Otolaryngology, Head and Neck Surgery, Leicester Royal Infirmary, Leicester, GBR; 2 Department of Intensive Care Unit and Anesthesia, Princess Alexandra Hospital, Harlow, GBR; 3 Department of Otolaryngology, Head and Neck Surgery, Naraina Medical College and Research Center, Kanpur, IND; 4 Department of Dentistry, Expert ENT and Diagnostic Center, Barabanki, IND; 5 Department of Otolaryngology, Head and Neck Surgery, Lancashire Teaching Hospitals NHS Foundation Trust, Preston, GBR; 6 Department of Otolaryngology, Head and Neck Surgery, Wrightington, Wigan and Leigh NHS Foundation Trust, Wigan, GBR

**Keywords:** aesthetic surgery, anthropometry, microtia repair and auricular reconstruction, otoplasty, pinna

## Abstract

Objectives

To assess the dimensions of external ear (pinna) in different age groups in the North Indian population. To assess the mean dimensions of external ear (pinna) in different age groups in North Indian males and females.

Methods

The study area was Lucknow/Barabanki, Uttar Pradesh, and the study center was Era's Lucknow Medical College, Uttar Pradesh, India. Study subjects were the study group, which consisted of 1807 subjects divided into six groups depending on age. Each group had a minimum of 300 subjects. Instruments used were digital vernier calipers, scale, and compass. Total ear height (TEH), lobular height (LH), lobular width (LW), ear projection (EP), ear width (EW), external auditory meatus length (EAML), external auditory meatus breadth (EAMB), distance from the tragus to the antihelix (DTA/TA), and distance from the tragus to the helix (DTH/TH).

Results

Statistically, no significant difference between the two sides was observed for any of the measurements. This shows that a bilateral symmetry was present, and hence, measurements of one side could be considered to be identical to that of the other. For subsequent representation, the average of two sides has been taken as the representative value of an individual. For group I, mean values±SD for measurement of TEH, LH, LW, EP, EW, EAML, EAMB, DTA, and DTH were 36.17±0.52, 5.38±0.33, 9.83±0.38, 5.53±0.46, 20.04±0.72, 2.00±0.49, 1.42±0.33, 12.55±0.45, and 18.63±0.44, respectively. For group II, mean values±SD for measurement of TEH, LH, LW, EP, EW, EAML, EAMB, DTA, and DTH were 47.29±4.53, 10.52±1.84, 14.93±1.37, 9.83±2.95, 25.83±3.08, 4.26±1.76, 3.02±1.78, 16.62±2.48, and 22.41±3.57, respectively. For group III, mean values±SD for measurement of TEH, LH, LW, EP, EW, EAML, EAMB, DTA, and DTH were 53.05±5.12, 13.36±1.76, 19.03±2.39, 17.57±3.03, 27.92±3.63, 6.82±2.03, 5.17±1.14, 17.81±1.83, and 23.71±1.97, respectively. For group IV, mean values±SD for measurement of TEH, LH, LW, EP, EW, EAML, EAMB, DTA, and DTH were 58.06±2.53, 16.63±1.65, 20.56±3.58, 19.47±1.55, 29.36±3.16, 8.64±1.28, 6.50±1.14, 17.75±1.31, and 24.83±1.70, respectively. For group V, mean values±SD for measurement of TEH, LH, LW, EP, EW, EAML, EAMB, DTA, and DTH were 60.24±3.46, 17.45±2.63, 22.07±3.65, 17.67±2.85, 30.21±3.96, 9.64±1.07, 7.04±0.94, 17.93±2.01, and 25.11±2.39, respectively. For group VI, mean values±SD for measurement of TEH, LH, LW, EP, EW, EAML, EAMB, DTA, and DTH were 64.88±4.04, 19.38±2.05, 22.15±2.54, 20.29±2.84, 33.66±2.17, 9.22±1.41, 7.07±1.20, 20.30±4.74, and 27.11±3.55, respectively.

Conclusions

On the basis of the above study, the utility of normative values can be discussed and explored further for the age and gender validation from the forensic point of view as well as from the point of view of prosthetic rehabilitation in different age groups and for the two genders.

## Introduction

Anthropometry refers to the measurement of the human individual. It has been used for identification, for the purpose of understanding human physical variations, and in various attempts to correlate physical with racial and psychological traits [1]. The external auricle or pinna develops from a series of cartilaginous tubercles that surround the first pharyngeal groove. Around the sixth week of embryonic life, a series of six tubercles appear around the first branchial arch; they enlarge and coalesce to form an auricle. It seems that the entire pinna, except for the tragus and anterior external auditory canal (of mandibular arch origin), arises from the hyoid (second branchial) arch [2]. The rudimentary pinna is formed by 60 days and achieves adult shape by the fifth month, although it apparently continues to grow throughout life. The ear is divided into three parts: the external ear (auricle), external auditory canal, middle ear, and inner ear. The external ear projects at variable angles from the side of the head and has some functions in collecting sounds. The lateral surface of the auricle has some prominences and depressions, which are different in every individual, even among identical twins. This unique pattern is comparable to fingerprints and can allow the identification of persons on the physiognomy of their auricles [3]. The external ear plays an important role in normal hearing. The outer ear functions to collect sound waves around us and funnels them to the eardrum through the ear canal. The concavities and convexities of the pinna interfere with sound and modify the spectrum. One may also describe the pinna as a form of audio filter. The funnel-like shape gives away another important function, i.e., the amplification of sound. Amplification of sound by the pinna, tympanic membrane, and middle ear causes an increase in level of about 10 to 15 dB in a frequency range of 1.5 kHz to 7 kHz [4,5]. Far from being vestigial, the complex structures of the pinna and external ear canal are now recognized as significant components in the mechanisms that underlie the capacity of a listener to recognize and localize sounds in space [6,7]. It also helps in the localization of sound and is provided by binaural interaction. The external ear provides important cues that are useful in monaural localization and, where binaural hearing is concerned, in enabling us to distinguish in front from behind and up from down [8].

The external ear is a critical component of the overall esthetic balance and contour of the face [9]. Its characteristic three-dimensional topography consists of interrelated length, width, and lateral projection, such that even slight alterations in the size, shape, location, or position of the ear are easily recognized, especially when compared with an opposite "normal" ear. Hence, better appearance and symmetry of the pinna are essential for facial harmony. Therefore, an anthropometric profile of the pinna is needed.

## Materials and methods

**Figure 1:**
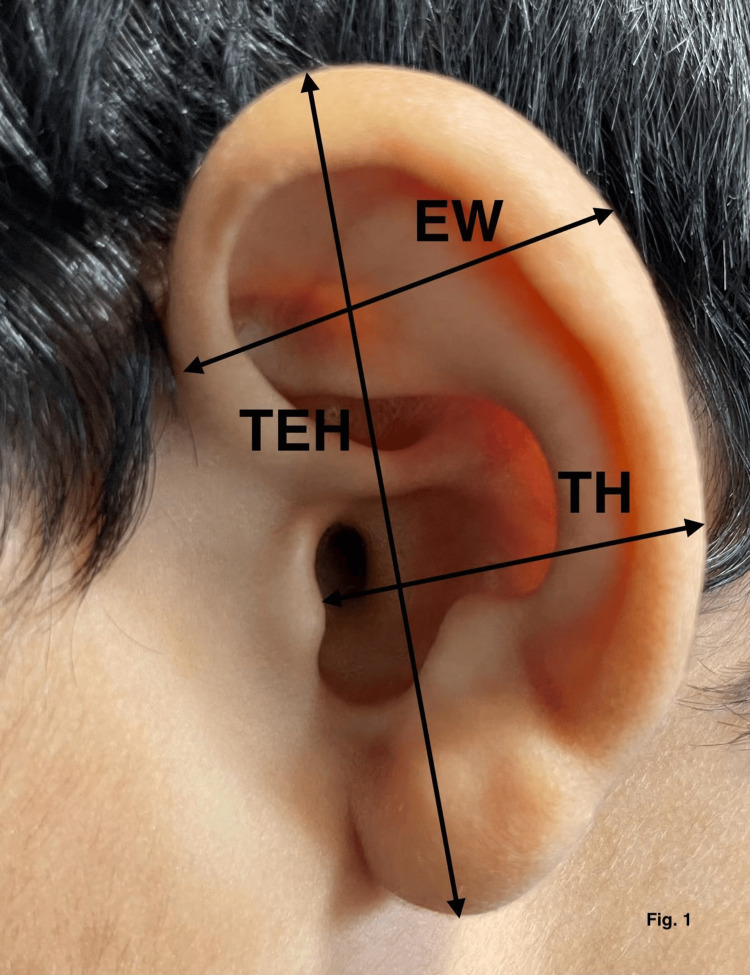
Morphometric measurements of total ear height (TEH) distance from tragus to helix (TH) and ear width (EW).

**Figure 2:**
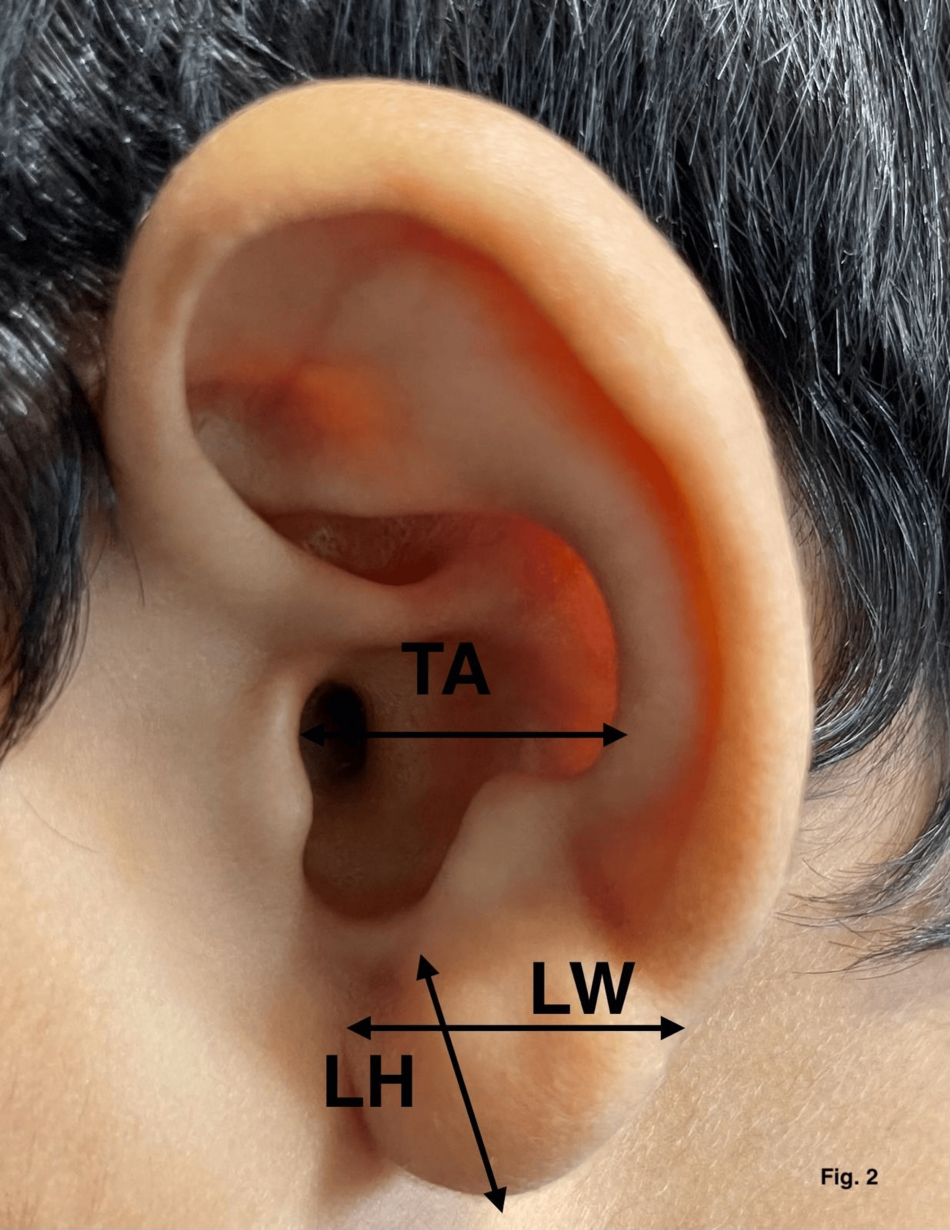
Morphometric measurements of lobular height (LH), lobular width (LW) and distance from tragus to antihelix (TA).

**Figure 3:**
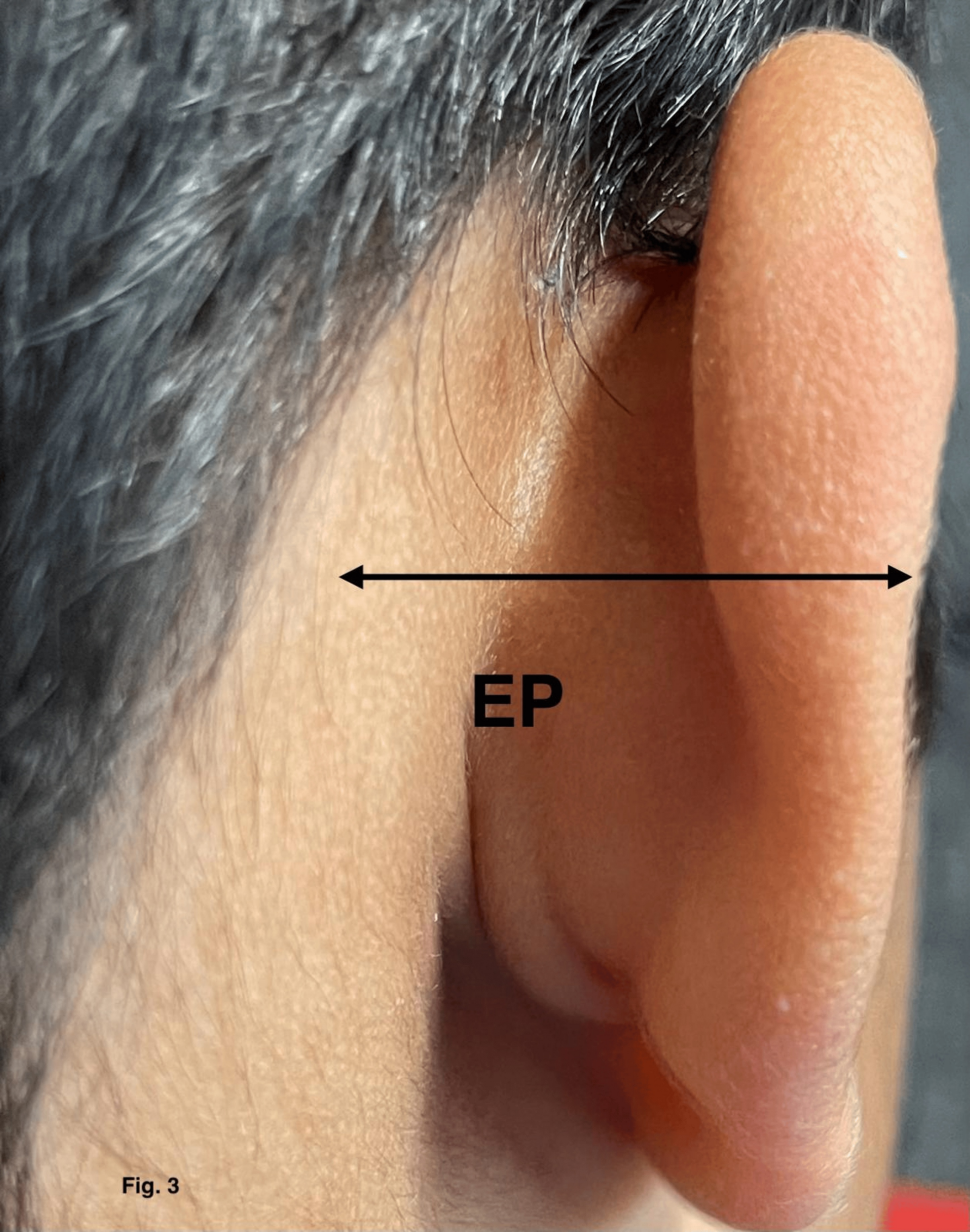
Morphometric measurement of ear projection (EP).

The study area was Lucknow/Barabanki, Uttar Pradesh, and the study center was Era's Lucknow Medical College, Uttar Pradesh, India. Study subjects were the study groups, which consisted of a total of 1807 subjects divided into six groups depending on age. Each group had a minimum of 300 subjects. Inclusion criteria include normal and healthy individuals of either sex and any age and only term newborn. Exclusion criteria include those with congenital abnormalities, preterm newborns, those suffering from an illness leading to physical deformities, and those who have incurred a traumatic injury leading to physical deformity. Study groups include group I: full-term neonates, group II: one month to five years, group III: six to 12 years, group IV: 13-17 years, group V: 18-50 years, and group VI: >50 years. Instruments used were digital vernier calipers, scale, and compass. After enrollment, nine surface measurements were taken directly from each ear of the subjects with an electronic digital caliper by the same investigator. These measurements were as follows in Table 1, Figures 1-3.

**Table 1:** All the measurements were made for both ears and were recorded in millimeters with a precision upto two decimal points.

Measurement	Description
Total ear height (TEH)	Distance between the highest point of auricle and lowest point of ear lobe
Lobular height (LH)	Distance from inter tragic incisure to the caudal part of lobule
Lobular width (LW)	Horizontal width of the lobule at the midpoint of the lobular height
Ear projection (EP)	Distance from the helix to the processus mastoideus at the tragal level
Ear width (EW)	Distance between the most anterior and posterior points of the ear
External auditory meatus length (EAML)	Length of external auditory meatus
External auditory meatus breadth (EAMB)	Breadth of external auditory meatus
Distance from the tragus to the antihelix (DTA/TA)	
Distance from the tragus to the helix (DTH/TH)	

Reliability 

Each measurement was done three times. An average of three measurements was taken as the final measurement. Data was analyzed using a Statistical Package for Social Sciences Version 20 (Released 2011; IBM Corp., Armonk, New York, USA). Descriptive analysis was performed to generate normative data. Values falling into 95% confidence interval (95% CI) levels were proposed to be used as normative values for the particular age and gender groups. The bilateral symmetry of the measurements was ascertained using a paired “t”-test. Comparison of measurements among different age groups was done using analysis of variance (ANOVA). Between gender comparisons were performed using independent samples “t” test. The confidence level of the study was kept at 95%. Hence, a “p” value less than 0.05 indicated a statistically significant difference.

Permission to carry out the study was obtained from the Institutional Ethical Committee. Informed consent was obtained from all the participants and their parents and guardians.

## Results

**Figure 4:**
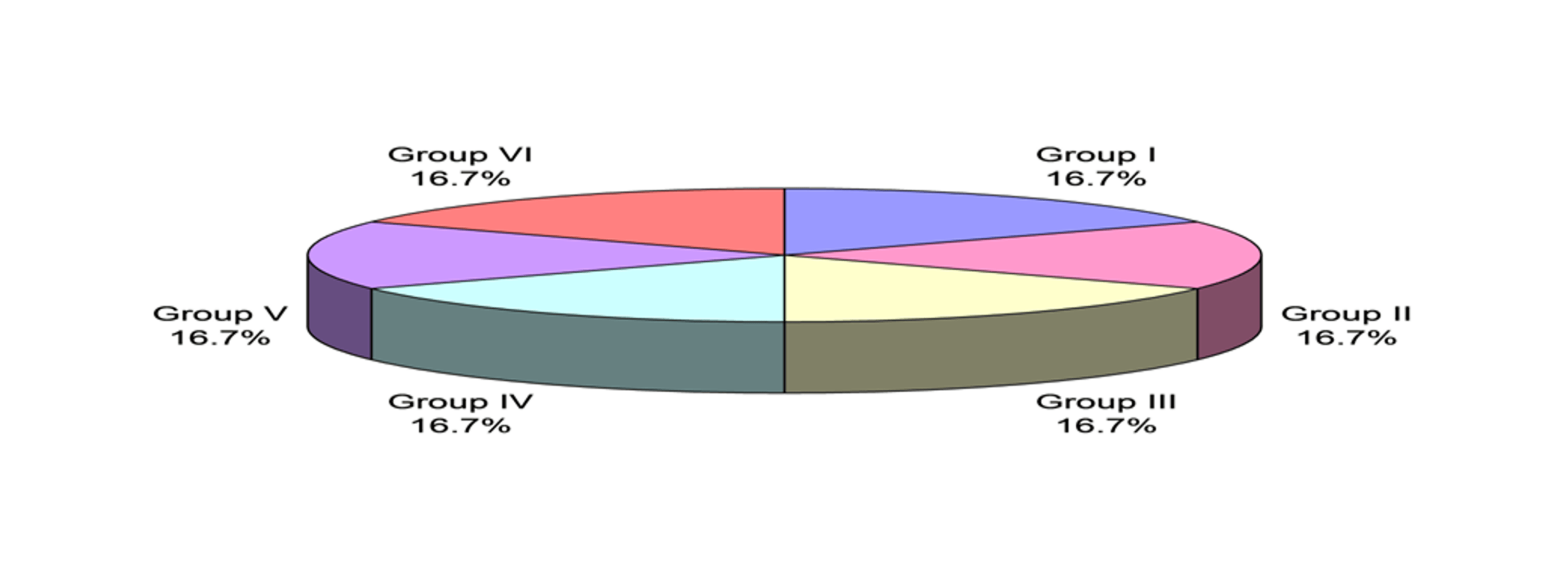
Group-wise distribution of subjects.

The present study was carried out with the aim of achieving the standardization of anthropometric values of the external ear (pinna) in different age groups of the North Indian population. For this purpose, a total of 1807 subjects, from freshly born neonates to those aged 72 years, were enrolled in the study. The subjects were divided into six study groups based on age (Figure 4, Table 2). 

**Table 2:** Descriptive statistics for external ear measurement to provide the basis for normative values in each age group. TEH: total ear height, LH: lobular height, LW: lobular width, EP: ear projection, EW: ear width, EAML: external auditory meatus length, EAMB: external auditory meatus breadth, DTA/TA: distance from the tragus to the antihelix, DTH/TH: distance from the tragus to the helix, SD: standard deviation, COV: coefficient of variance, FT: full term.

Variable	Mean	SD	COV	95% CI		Min	Max
				Lower	Upper		
Group I (FT) (n=302)							
TEH	36.17	0.52	0.01	36.11	36.23	35.21	37.23
LH	5.38	0.33	0.06	5.35	5.42	5.01	5.98
LW	9.83	0.48	0.05	9.77	9.88	9.10	10.59
EP	5.53	0.46	0.08	5.48	5.58	4.78	6.09
EW	20.04	0.72	0.04	19.96	20.12	19.01	20.99
EAML	2.00	0.49	0.25	1.94	2.06	1.14	3.01
EAMB	1.42	0.33	0.24	1.39	1.46	1.02	1.99
DTA	12.55	0.45	0.04	12.50	12.60	11.74	12.99
DTH	18.63	0.44	0.02	18.58	18.68	18.02	19.43
Group II (1-5 years)							
TEH	47.29	4.53	0.10	46.78	47.81	40.02	52.65
LH	10.52	1.84	0.17	10.31	10.73	7.17	13.50
LW	14.93	1.37	0.09	14.78	15.09	12.09	16.99
EP	9.83	2.95	0.30	9.49	10.16	6.01	14.03
EW	25.83	3.08	0.12	25.48	26.18	21.04	31.96
EAML	4.26	1.76	0.41	4.06	4.46	2.97	8.91
EAMB	3.02	1.78	0.59	2.82	3.22	1.91	7.78
DTA	16.62	2.48	0.15	16.34	16.90	12.87	20.11
DTH	22.41	3.57	0.16	22.01	22.82	17.11	27.18
Group III (6-12 years)							
TEH	53.05	5.13	0.10	52.47	53.63	43.19	58.58
LH	13.36	1.76	0.13	13.16	13.56	10.14	15.54
LW	19.03	2.39	0.13	18.76	19.30	12.86	21.12
EP	17.57	3.03	0.17	17.23	17.92	13.57	22.86
EW	27.93	3.63	0.13	27.52	28.34	24.19	33.96
EAML	6.83	2.02	0.30	6.60	7.06	3.12	9.65
EAMB	5.17	1.14	0.22	5.04	5.30	2.98	6.68
DTA	17.82	1.83	0.10	17.61	18.03	14.40	20.49
DTH	23.71	1.96	0.08	23.49	23.93	20.32	26.56
Group IV (13-17 years)							
TEH	58.06	2.53	0.04	57.78	58.35	53.19	61.55
LH	16.63	1.65	0.10	16.44	16.82	14.14	22.75
LW	20.56	3.58	0.17	20.15	20.96	15.49	25.98
EP	19.47	1.55	0.08	19.30	19.65	17.98	22.87
EW	29.36	3.16	0.11	29.00	29.72	24.03	33.82
EAML	8.64	1.28	0.15	8.50	8.79	6.89	10.89
EAMB	6.50	1.14	0.17	6.37	6.63	4.99	8.79
DTA	17.75	1.31	0.07	17.60	17.89	15.21	20.64
DTH	24.83	1.70	0.07	24.64	25.03	21.58	27.55
Group V (18-50 years)							
TEH	60.24	3.46	0.06	59.85	60.64	53.54	69.61
LH	17.45	2.63	0.15	17.15	17.75	11.06	23.30
LW	22.07	3.65	0.17	21.66	22.48	13.45	28.08
EP	17.67	2.85	0.16	17.35	18.00	11.09	25.11
EW	30.21	3.96	0.13	29.76	30.66	23.12	36.67
EAML	9.64	1.07	0.11	9.52	9.76	7.49	11.95
EAMB	7.04	0.94	0.13	6.93	7.14	5.12	9.05
DTA	17.93	2.01	0.11	17.70	18.16	13.87	23.43
DTH	25.11	2.39	0.10	24.84	25.38	20.66	29.89
Group VI (>50 years)							
TEH	64.88	4.04	0.06	64.42	65.34	60.17	73.01
LH	19.38	2.05	0.11	19.15	19.62	15.75	21.62
LW	22.15	2.54	0.11	21.87	22.44	18.04	25.87
EP	20.29	2.84	0.14	19.97	20.62	14.35	23.69
EW	33.66	2.17	0.06	33.41	33.90	29.76	36.49
EAML	9.22	1.41	0.15	9.06	9.38	7.30	11.89
EAMB	7.07	1.20	0.17	6.93	7.20	5.37	9.05
DTA	20.30	4.74	0.23	19.76	20.84	14.99	28.87
DTH	27.11	3.55	0.13	26.71	27.51	22.43	34.50

Mean external ear measurements in group I 

For group I, mean values±SD for measurement of TEH, LH, LW, EP, EW, EAML, EAMB, DTA, and DTH were 36.17±0.52, 5.38±0.33, 9.83±0.38, 5.53±0.46, 20.04±0.72, 2.00±0.49, 1.42±0.33, 12.55±0.45, and 18.63±0.44 respectively. The coefficient of variance (COV) was minimum for TEH 0.01, thus indicating maximum consistency, while it was maximum for EAML and EAMB (0.25 and 0.24, respectively), thus indicating minimum consistency. The normative range (95%CI) for TEH was 36.11 to 36.23, whereas the same for EAML and EAMB was 1.94 to 2.06 and 1.39 to 1.46, respectively. For LH, LW, EP, EW, DTA, and DTH, the normative range (based on 95% confidence limits) was 5.35-5.42, 9.77-9.88, 5.48-5.88, 19.96-20.12, 12.50-12.60 and 18.58-18.68, respectively. It was observed that for all the measurements, the normative range was in close proximity to the mean values.

Mean external ear measurements in group II 

For group II, mean values±SD for measurement of TEH, LH, LW, EP, EW, EAML, EAMB, DTA, and DTH were 47.29±4.53, 10.52±1.84, 14.93±1.37, 9.83±2.95, 25.83±3.08, 4.26±1.76, 3.02±1.78, 16.62±2.48, and 22.41±3.57, respectively. The coefficient of variance (COV) was minimum for EP (COV=0.09), thus indicating maximum consistency, while it was maximum for EAMB (COV=0.59), thus indicating minimum consistency. The normative range (95%CI) for TEH, LH, LW, EP, EW, EAML, EAMB, DTA and DTH was 46.78-47.81, 10.31-10.73, 14.78-15.09, 9.49-10.16, 25.48-26.18, 4.06-4.46, 2.82-3.22, 16.34-16.90 and 22.01-22.82, respectively. It was observed that for all the measurements, the normative range was in close proximity to the mean values.

Mean external ear measurements in group III 

For group III, mean values±SD for measurement of TEH, LH, LW, EP, EW, EAML, EAMB, DTA, and DTH were 53.05±5.12, 13.36±1.76, 19.03±2.39, 17.57±3.03, 27.92±3.63, 6.82±2.03, 5.17±1.14, 17.81±1.83, and 23.71±1.97, respectively. The coefficient of variance (COV) was minimum for DTH (COV=0.08), thus indicating maximum consistency, while it was maximum for EAML (COV=0.30), thus indicating minimum consistency. The normative range (95%CI) for TEH, LH, LW, EP, EW, EAML, EAMB, DTA, and DTH was 52.47-53.63, 13.16-14.56, 18.76-19.30, 17.23-17.92, 27.51-28.34, 6.59-7.05, 5.04-5.30, 17.60-18.01, and 23.49-23.93, respectively. It was observed that for all the measurements, the normative range was in close proximity to the mean values.

Mean external ear measurements in group IV 

For group IV, mean values±SD for measurement of TEH, LH, LW, EP, EW, EAML, EAMB, DTA, and DTH were 58.06±2.53, 16.63±1.65, 20.56±3.58, 19.47±1.55, 29.36±3.16, 8.64±1.28, 6.50±1.14, 17.75±1.31, and 24.83±1.70, respectively. The coefficient of variance (COV) was minimum for TEH (COV=0.04), thus indicating maximum consistency, while it was maximum for LW and EAML (COV=0.17), thus indicating minimum consistency. The normative range (95%CI) for TEH, LH, LW, EP, EW, EAML, EAMB, DTA, and DTH was 57.78-58.35, 16.44-16.82, 20.15-20.96, 19.30-19.65, 29.00-29.72, 8.50-8.79, 6.37-6.63, 17.60-17.89, and 24.64-25.03, respectively. It was observed that for all the measurements, the normative range was in close proximity to the mean values.

Mean external ear measurements in group V 

For group V, mean values±SD for measurement of TEH, LH, LW, EP, EW, EAML, EAMB, DTA, and DTH were 60.24±3.46, 17.45±2.63, 22.07±3.65, 17.67±2.85, 30.21±3.96, 9.64±1.07, 7.04±0.94, 17.93±2.01, and 25.11±2.39, respectively. The coefficient of variance (COV) was minimum for TEH (COV=0.06), thus indicating maximum consistency, while it was maximum for LW (COV=0.17), thus indicating minimum consistency. The normative range (95%CI) for TEH, LH, LW, EP, EW, EAML, EAMB, DTA, and DTH was 59.85-60.64, 17.15-17.75, 21.66-22.48, 17.35-18.00, 29.76-30.66, 9.52-9.76, 6.93-7.14, 17.70-18.16 and 24.84-25.38, respectively. It was observed that for all the measurements, the normative range was in close proximity to the mean values.

Mean external ear measurements in group VI 

For group VI, mean values±SD for measurement of TEH, LH, LW, EP, EW, EAML, EAMB, DTA, and DTH were 64.88±4.04, 19.38±2.05, 22.15±2.54, 20.29±2.84, 33.66±2.17, 9.22±1.41, 7.07±1.20, 20.30±4.74, and 27.11±3.55, respectively. The coefficient of variance (COV) was minimum for TEH and EW (COV=0.06), thus indicating maximum consistency, while it was maximum for DTA (COV=0.23), thus indicating minimum consistency. The normative range (95%CI) for TEH, LH, LW, EP, EW, EAML, EAMB, DTA, and DTH was 64.42-65.34, 19.15-19.62, 21.87-22.44, 19.97-20.62, 33.41-33.90, 9.06-9.38, 6.93-7.20, 19.76-20.84, and 26.71-27.51, respectively. It was observed that for all the measurements, the normative range was in close proximity to the mean values.

Age-wise differences

(a) TEH: An increase in TEH was observed with increasing age. Statistically, the association between age and TEH length was significant too (p<0.001). (b) LH: With increasing age, a significant increase in the mean value of LH was observed (p<0.001). (c) LW: With increasing age, an increasing trend in mean LW values was observed, which was also significant statistically (p<0.001). It was observed that the increase showed a steep from group I to group IV; however, in groups V and VI, a static trend was observed. (d) EP: For EP, an increasing trend of mean values was observed from group I to group IV; however, in group V, mean values showed a decline followed by a rise to reach the maximum level in group VI (p<0.001). (e) EW: For EW, a significant increase in mean values was observed with increasing age (p<0.001). The mean value was minimum in group I and maximum in group VI. (f) EAML: For EAML, an increasing trend was observed from group I to group V; however, in group VI, a decline in mean values was observed. Statistically, there were significant differences in mean EAML values among the groups (p<0.001). Mean values were minimum in group I and maximum in group V. (g) EAMB: For EAMB, mean values showed an increasing trend with increasing age, which was also significant statistically (p<0.001). A steep increase in mean values was observed from group I to group V. However, in group V, the mean values showed a statistical trend. (h) DTA/TA: A steep rise in DTA values was observed from group I to group III; however, from group III to group V, the values remained almost static, followed by a sudden rise in group VI. Statistically, these differences among groups were significant (p<0.001). (i) DTH/TH: An increasing trend in mean DTH values was observed with increasing age. The growth was accelerated from group I to III, followed by a static trend till group V; once again, a growth spurt was observed between group V and group VI stages. Statistically, differences among groups were significant (p<0.001).

## Discussion

The ear is one of the most important sensory organs of the human body. Considering the sensory and esthetic functions, external ears are assumed to have an important role in human anthropometry, and their deviation from a definite proportion and size might affect the sensory and esthetic functions. It is an essential part of a person's physical appearance and hence can influence other persons' perceptions about an individual's personality [10]. Despite its functional and esthetic significance, there is a lack of sufficient literature on the morphometry of the external ear in different population groups, especially that of the Indian population. Hence, the present study was planned to study the morphological characteristics of external ears in the Indian population for both genders and at different life stages. Evaluations were done among different age groups. The purpose was to understand the age-wise differences in morphological parameters and the chronology of growth and change in the morphology of the external ear. An attempt was also made to compare the bilateral symmetry of two ears.

Bilateral symmetry

Mean differences in measurements of different parameters at two sides ranged from 0.000 to 0.005 mm. Statistically, this difference was not significant for any of the parameters (p>0.05). Bilateral asymmetry of the face was investigated in detail. One interesting contribution of this research was the idea of a “truthful” vertical axis for the face and a special virtual coordinate system for the eye region. Another was the analysis of various facial components and their effect on face symmetry [11]. The quantitative measurements in the present study revealed the existence of bilateral symmetry, thus indicating that the normative values for different parameters of one side can successfully be used for the other side, too. 

Age-wise normative values

Patients were divided into six age groups. The 95% confidence interval range was used for the normative range to rule out the effect of extremes and outliers. A study of change patterns in morphometry of different parameters revealed a continuous age-associated change in external ear dimensions in different planes. This inference is in agreement with the observations, which have also supported the view that the age-associated changes in auricular size continue during adulthood [12]. However, to say that the rate and pattern of growth for different parameters were similar across all the life stages is not appropriate, as the rate and pattern of growth in different planes and at different life stages varied considerably. It was observed that ear length reaches around 90% of mature size during the adolescence age (13-18 years) [13,14]. In the present study, we observed that the total ear length achieved was around 87% of the total ear length of subjects who had attained adulthood (18-50 years), thus showing a similar growth trend as observed by the other workers. However, the additional finding in the present study was that the growth continued even after attaining adulthood, though at a slower rate. Most of the studies in the literature are limited to 18 years of age [13,14] and thus have limitations in exploring this aspect. In the present study, we also observed a growth pattern throughout life, but it was at different rates at different stages of life and did not follow a mathematical trend. The present study is perhaps the first attempt in North India to exhaustively study the growth pattern of external ears in different age groups and both genders for a total of ten morphometric parameters and to come up with a normative data set for the North Indian population. The findings bring forth the need for separate normative data for different age groups and for both genders, given the fact that there were significant gender differences in almost all age groups. The findings provide a basis for future work, especially for forensic sciences and prosthesis of ears in different age groups and for different genders.

## Conclusions

On the basis of observations made in the present study, the following findings were deduced: Age-wise differences in normative values of different parameters were observed. An increasing trend of measurements was observed with increasing age. That is why age-specific normative ranges have been proposed for all the parameters. For the entire population, a coefficient of variance less than 0.05 (5%) was observed for 4/9 parameters in group I, 0/9 in groups II, III, V, and VI, and 1/9 in group IV, thus indicating that normative values were most effectively applicable in newborns. On the basis of the above study, the utility of normative values can be discussed and explored further for age and gender validation from the forensic point of view as well as from the point of view of prosthetic rehabilitation in different age groups and for the two genders.
